# Fractographic Analysis and Fatigue Crack Propagation Behavior of TC4-F Alloy with a Duplex Microstructure

**DOI:** 10.3390/ma19112238

**Published:** 2026-05-25

**Authors:** Yangyang Sun, Li Liu, Zhongyang Mao, Feifei Jiang, Lian Zhou

**Affiliations:** 1 College of Civil Engineering, Nantong Institute of Technology, Nantong 226601, China; sunyangyang0526@163.com (Y.S.);; 2 College of Materials Science and Engineering, Nanjing Tech University, Nanjing 210009, China; 3 Wisdom Library, Nantong Institute of Technology, Nantong 226601, China

**Keywords:** titanium alloy, fatigue crack propagation, fracture morphology, trace Fe

## Abstract

The fatigue performance of titanium alloys is a critical determinant of the service life and structural integrity for aerospace and marine engineering components. But within the framework of damage tolerance design, resistance to fatigue crack propagation is regarded as a key indicator governing the fatigue performance of these engineering structures. In previous work, while the general fatigue performance of Ti–6Al–4V-0.55Fe alloy has received systematic study, targeted research focusing on its resistance to fatigue crack propagation remains limited. Therefore, in this work, compared with Ti–6Al–4V ELI alloy, the fatigue crack propagation behavior and fracture mechanism of Ti–6Al–4V-0.55Fe alloy with a duplex microstructure were systematically investigated. The results show that when ∆*K* < 12.75 MPa⋅m^1/2^, Ti-6Al-4V-0.55Fe alloy demonstrates superior resistance to fatigue crack propagation. Fractographic analysis indicates that the primary difference between the two alloys lies in the stage of crack initiation and early propagation. This behavior is attributed to the addition of trace Fe, which enhances α/β boundary resistance and thereby retards crack growth. Moreover, crack propagation of TC4-F alloy is also slowed by the increased path length from bypassing the α_p_ phase.

## 1. Introduction

Distinguished by its exceptional specific strength, corrosion resistance, and high-temperature mechanical stability [1,2], TC4 (Ti-6Al-4V) titanium alloy has become an indispensable key structural material in aerospace, biomedical, and marine engineering fields [3,4,5]. In critical applications such as aero-engine fan disks, compressor blades, and fuselage load-bearing structures, TC4 components are always subjected to prolonged complex alternating loads, which could cause the fatigue fracture. Under this condition, the fatigue resistance of this material is the primary determinant of overall equipment reliability and operational life. Therefore, enhancing the fatigue resistance of TC4 alloys without reducing the tensile properties has become an urgent issue that researchers need to address. Under this background, based on the alloying element partitioning effect, the Ti-6Al-4V-0.55Fe alloy with better tensile and fatigue properties was developed [6].

Up to now, a substantial body of research have been accumulated on the fatigue crack of TC4 alloy. Authors such as Chen et al. [7] investigated the fatigue crack initiation mechanism of TC4 alloy during fatigue deformation and proposed a crystal plasticity finite element method, varying the volume fractions of the transformed β phase to clarify the fatigue crack initiation process. Kuang et al. [8] found that the increase in fatigue resistance of TC4 alloy was credited with activated cross-slip and deformation twinning, which delayed the crack initiation. Junet et al. [9] used synchrotron X-ray tomography and obtained a result showing that internal fatigue cracks of TC4 alloy tended to propagate at the notch in two distinct propagation regimes, while Ueki et al. [10] systematically emphasized the impact of the crystallographic orientation and lamellar configuration on fatigue crack propagation of TC4 alloy, and a detailed analysis of their crack propagation and fatigue damage mechanisms was explored. Gao et al. [11] emphasized the fact that the stress intensity factor range (Δ*K*) serves as the primary driving force governing fatigue crack growth in titanium alloy, acting as a critical parameter for fitness-for-service and damage tolerance evaluation. Strictly, the focus of traditional safe-life design is the fatigue property, which emphasizes the initiation of failure [12]. Conversely, damage tolerance design is concerned with the crack propagation process, with the goal of enhancing the operational lifespan of components [13].

Based on the TC4 alloy, many research studies have also been conducted on the fatigue resistance of Ti-6Al-4V-0.55Fe alloy. Our early work [6] was the first to pay attention to the low-cycle fatigue behavior and fracture characteristics of the rolled alloy, and we analyzed the softening behavior of the alloy from the perspective of internal stress. Moreover, the effects of Fe on sliding resistance, boundary resistance, and fatigue crack propagation have been preliminarily analyzed and understood. However, a systematic comparison of crack propagation behavior between the base alloy and the Fe-modified alloy has not yet been conducted. Shi et al. [14] focused on the low-cycle dwell fatigue behavior and microstructure evolution. The effect of microstructure, the corrosive environment, the control of strain and stress amplitudes on fatigue behavior and the deformation mechanism have been thoroughly studied through theoretical research and supplemented with data in previous works [15,16,17]. Moreover, in terms of the driving force for fatigue crack initiation, the partitioning effect of Fe element on cyclic deformation response and micro-mechanisms of this alloy has been profoundly revealed in a recent study [18]. In general, substantial understanding has been gained regarding the fatigue behavior and cyclic deformation mechanisms of alloys.

Since fatigue resistance dominates the service life management of engineering components, it is essential to reveal the influence mechanism of microalloying elements. However, there has been relatively little focus on the effect of Fe addition and fatigue crack propagation behavior of Ti-6Al-4V-0.55Fe alloy in the near-threshold stage. Therefore, in this work, through a systematic comparison with the Ti-6Al-4V ELI alloy, the mechanisms governing crack initiation and propagation of Ti-6Al-4V-0.55Fe alloy were elucidated, and the impact of trace Fe addition on the fracture characteristics and the suppression of crack propagation within the Ti-6Al-4V-0.55Fe alloy was also clarified. The results of this work could provide a scientific basis for optimizing damage tolerance strategies for this alloy.

## 2. Materials and Methods

### 2.1. Material

The rolled Ti-6Al-4V-0.55Fe (hereafter referred to as TC4-F) alloy plate with a thickness of 42 mm produced by vacuum arc remelting (VAR) was employed in this study. In order to systematically research the fatigue crack propagation behavior of this new alloy, the typical TC4 ELI (Ti-6Al-4V ELI) alloy produced by the same manufacturing procedure was also employed in this paper. Their chemical compositions are listed in Table 1. Based on the metallographic analysis, their β-transus temperatures (T_β_) are 975(5) °C and 950(5) °C. The initial materials are typically equiaxed microstructure. For achieving the duplex microstructure, solution treatment at 940 °C for 1.5 h with air cooling, and then aging at 580 °C for 4 h with air cooling, were adopted. Room temperature tensile properties of alloys have been described in previous research [18], and the TC4 F alloy exhibits higher yield strength (883.7 MPa) and elongation (16.6%) than that of TC4 ELI alloy (843.5 MPa, 15.7%).

### 2.2. Fatigue Crack Propagation Tests

**Figure 1:**
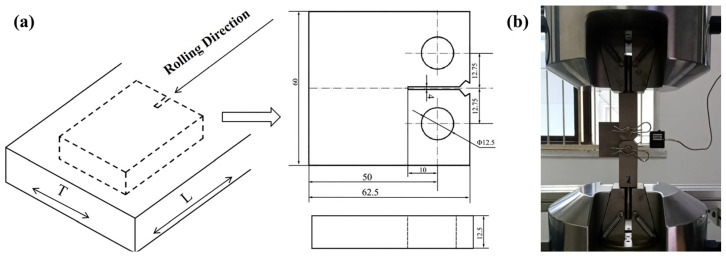
(**a**) Compact tensile specimen size diagram; (**b**) MTS 810 high-frequency fatigue testing machine.

The adopted standard compact tension (CT) samples, 12.5 mm thick and 60 mm wide, prepared by normative T-L direction are displayed in the schematic diagram of Figure 1. The fatigue crack propagation (FCP) tests of alloys were performed on MTS 810 high-frequency fatigue testing machine (MTS Co., Shanghai, China) at room temperature with a loading frequency of 15 Hz, a stress ratio R of 0.1, and a maximum load of 7.5 kN, in accordance with the relevant requirements of GB/T 6398-2017 [19]. The relevant data, such as fatigue crack length (a) and cycle number (N) could be obtained from tests. Accordingly, the crack propagation rate (d*a*/d*N*) and stress intensity factor range (Δ*K*) were calculated by the system. The calculation formula for computing the values of the Δ*K* is taken from [20,21]:

(1) $$\Delta K=\;\frac{∆P}{B\sqrt{W}}\;\frac{\left(2+\alpha \right)}{{\left(1-\alpha \right)}^{3/2}}\;(0.886+4.64\alpha -13.32\alpha^{2}+14.72\alpha^{3}-5.6\alpha^{4})$$

where *α* = *a*/*W*, *a* is the crack length, and *W* and *B* are the width and thickness of the samples, respectively. Δ*P* is the range of the applied load (∆*P* = *P_max_* − *P_min_*).

### 2.3. Microstructure Characterization

The microstructure observation and the fractographic morphology for alloys were examined by optical microscope (Olympus GX51, Ruike Co., Beijing, China) and scanning electron microscopy (SEM, JSM-6510, JOEL Co., Beijing, China).

## 3. Results

### 3.1. Microstructure

**Figure 2:**
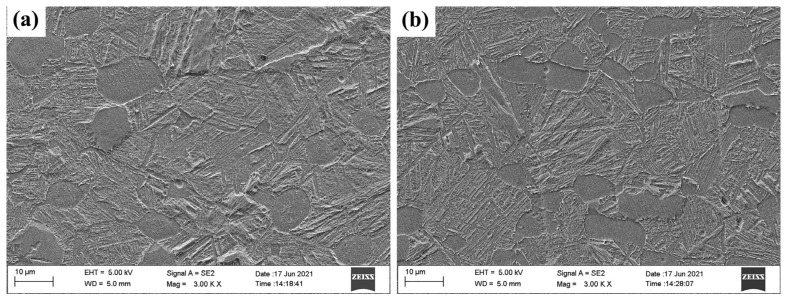
SEM image of the duplex microstructure: (**a**) TC4-F alloy; (**b**) TC4 ELI alloys [18].

Figure 2 shows the SEM images of duplex microstructure for TC4-F and TC4 ELI alloys. It is seen that the microstructure is composed of primary equiaxed α phase (α_p_), secondary α (α_s_), and β matrix (β_m_). In contrast, TC4 F has a smaller aspect ratio, of α_p_ (1~3.01), a lower volume fraction, of α_p_ (26.63%), and finer average thickness, of α_s_ (0.53 μm) [18].

### 3.2. Fatigue Crack Propagation Rates

**Figure 3:**
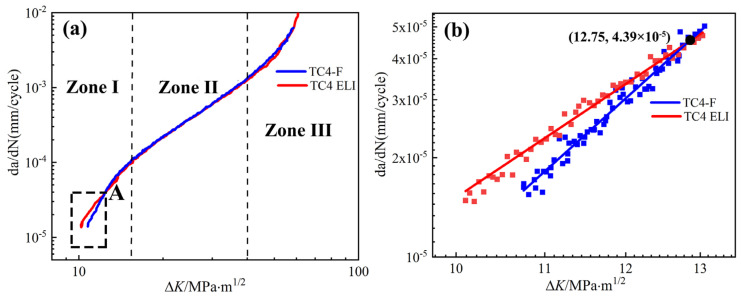
FCP rate curves of TC4-F and TC4 ELI alloys with duplex microstructure: (**a**) the overall FCP rate curves of the two alloys; (**b**) the local enlarged view of (**a**).

The FCP rate at R = 0.1 for TC4-F and TC4 ELI alloys are extracted, and shown in Figure 3. As shown in Figure 3a, the d*a*/d*N* curves of TC4 F and TC4 ELI alloys can be divided into three parts, which reflect the different stages of crack propagation [22,23], respectively. (i) Zone I: d*a*/d*N* is quite low, which is usually regarded as the fatigue crack non-propagation zone or the zone close to the threshold. (ii) Zone II: d*a*/d*N* shows a linear trend with ∆*K*, which has been reported as the subcritical fatigue crack growth stage or Paris zone. (iii) Zone III: d*a*/d*N* increases sharply, leading to fracture, which is commonly referred to as the transient fracture zone. And, among these, the FCP regime constitutes the majority of the remaining fatigue life [24]. Based on the linear fitting results specified by the test standard, the following Paris formula [25], which articulated the correlation between d*a*/d*N* and Δ*K*, could be obtained.

TC4-F alloy: d*a*/d*N* = 5.583 × 10^−8^ (∆*K*)^2.733^ (2)

TC4 ELI alloy: d*a*/d*N* = 5.189 × 10^−8^ (∆*K*)^2.765^ (3)

Moreover, it can be seen from Figure 3 that variation patterns of the d*a*/d*N* with ∆*K* are generally quite similar for both of the alloys, but their curves intersect at point A (12.75 MPa·m^1/2^, 4.39 × 10^−5^ mm/cycle). When ∆*K* > 12.75 MPa·m^1/2^, the FCP curves of the two alloys are basically superimposed. When ∆*K* < 12.75 MPa·m^1/2^, the TC4-F alloy has a relatively low crack propagation rate, with ∆*K* increasing. Since the initial stage of crack propagation accounts for the majority of the total propagation period, the lower crack propagation rate indicates that TC4-F alloy has a better fatigue life than that of TC4 ELI alloy (shown in Figure 3b). Accordingly, Δ*K* of 12.75 MPa·m^1/2^ can be seen as the critical point of the stress intensity factor range for TC4-F and TC4 ELI alloys. The different FCP behavior can be further analyzed by observing the morphological characteristics of the fracture surface.

### 3.3. Fracture Characteristics

**Figure 4:**
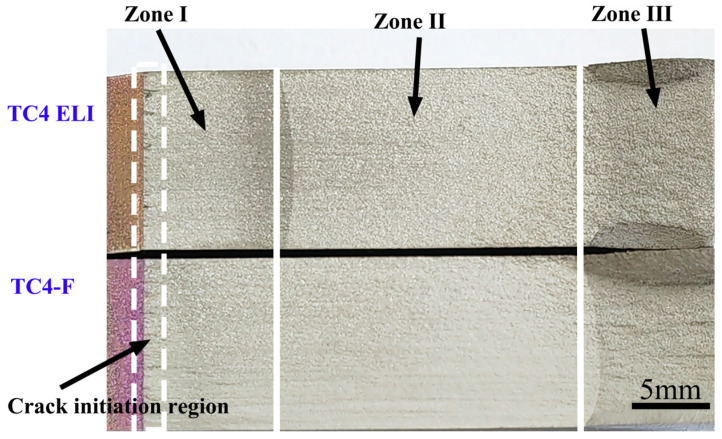
Macro-fracture morphologies images of d*a*/d*N* samples for TC4 F and TC4 ELI alloys.

In order to reveal the FCP behavior of TC4-F and TC4 ELI alloys under different stages, the macro-fracture morphologies of the fractured samples are observed in Figure 4 by the camera, respectively, and the divisions marked on the fracture are consistent with those shown in Figure 3. By analyzing the fracture morphologies of the two alloys, it can be observed that in the crack initiation region, multiple fatigue cracks start from the surface of the sample, presenting a pattern resembling a short river. As the crack propagation rate increases, the fracture surface becomes brighter, smoother and flatter. However, near zone III, the surface becomes rough again and exhibits morphological characteristics similar to those of static tensile testing.

Based on Formula (1), for TC4-F and TC4 ELI alloys the corresponding crack length and propagation length are determined as approximately 15 mm and 5 mm at the Δ*K* of 12.75 MPa·m^1/2^, respectively (Figure 3b). This indicates that the difference in crack propagation behavior between TC4-F and TC4 ELI alloys mainly lies in the early stage of FCP.

Fatigue failure of metallic materials often begins with the initiation and initial propagation of cracks, which account for the majority of the entire fatigue life [26]. This also is the main reason for the difference in FCP rate curves between TC4-F and TC4 ELI alloys.

**Figure 5:**
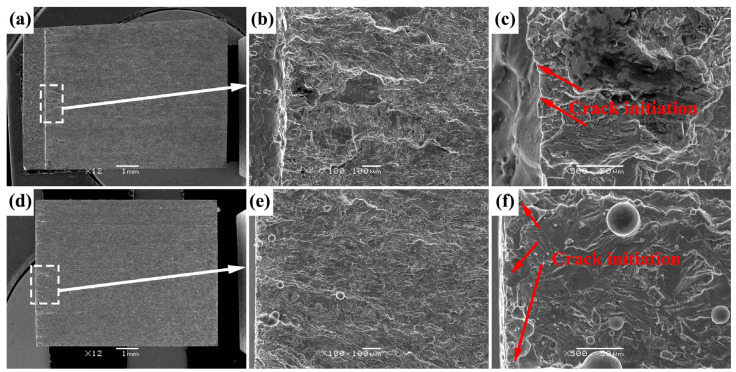
Fractography of pre-crack region: (**a**,**d**) macro-morphologies for TC4-F and TC4 ELI alloys, respectively; (**b**,**c**) local micro-topographies for TC4-F alloy; (**e**,**f**) local micro-morphologies for TC4-ELI alloy.

Therefore, in order to understand the crack initiation mechanism of the two alloys, the fracture morphologies of pre-crack regions (Figure 4) are observed by using SEM, as shown in Figure 5. The fracture of TC4-F alloy is relatively rough, with numerous tearing edges, small cleavage facets and cleavage steps extending along the crack propagation, which show the fracture characteristics of micro-cleavage as a whole. The formation of fatigue cracks generally includes the initiation, growth and connection of microcracks. The initiation of microcracks mainly involves three mechanisms: cracking along the surface slip bands, cracking at the interface between inclusions and the matrix, and cracking at the grain boundaries. It can be found from Figure 5b,c that the fatigue microcrack initiation of TC4-F alloy is dominated by slip band cracking. Under sufficient cyclic loads, the slip bands at the original grain boundaries or at small defects are activated. However, the different slip back stresses on slip planes lead to the asymmetry of slip, thereby making the sample surface rough. It shows “extrusion” peaks and “intrusion” valleys [27], which also indicates that the slip under cyclic loading is not completely reversible. From Figure 5d, it can be observed that the fracture surface of TC4 ELI alloy is relatively flat. This is because the microcracks initially originated and grew at the grain boundaries without undergoing significant deflection.

In order to deeply analyze the fatigue crack propagation mechanism of TC4-F alloy, compared with the TC4 ELI alloy, based on the fracture zone division shown in Figure 4, the fracture surface morphologies of Zone I, Zone II and Zone III were successively observed.

(1) Zone I

**Figure 6:**
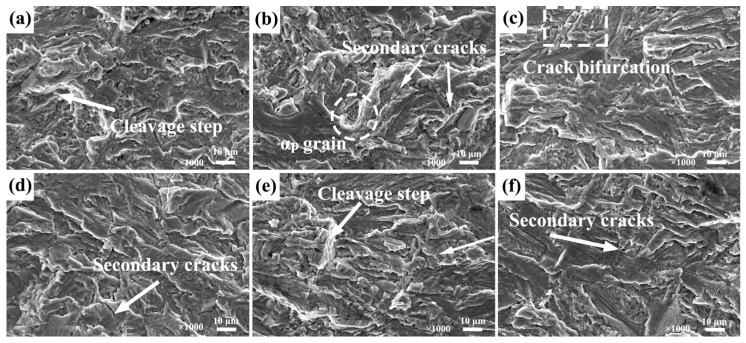
Fractographies of Zone I: (**a**–**c**) TC4-F alloy; (**d**–**f**) TC4 ELI alloy.

The fractographies of selected position, close to the turning point A on the FCP rate curves (Figure 3) for the two alloys, are illustrated by Figure 6. At this stage, the stress intensity factor range Δ*K* is still relatively low, with the process from no crack to plastic deformation and then to crack propagation. The plastic zone size at the crack tip is relatively small, and it may still be in the state of plane strain. It can be seen from Figure 6 that the fracture morphologies of the two alloys are quite similar, and the cleavage steps and secondary cracks are observed, which belong to the typical cleavage or quasi-cleavage fracture characteristics. The similarity in the overall fracture surfaces reflects the macro-mechanism of crack propagation. While crack initiation sites differ (Figure 5), due to the effect of localized microstructure and dislocation movement, the final mode of fracture converges, which is attributable to the shared material properties for the two alloys. Moreover, intergranular fracture (Figure 6b) and crack bifurcation (Figure 6c) are also observed on the fracture surface of TC4-F alloy.

(2) Zone II

**Figure 7:**
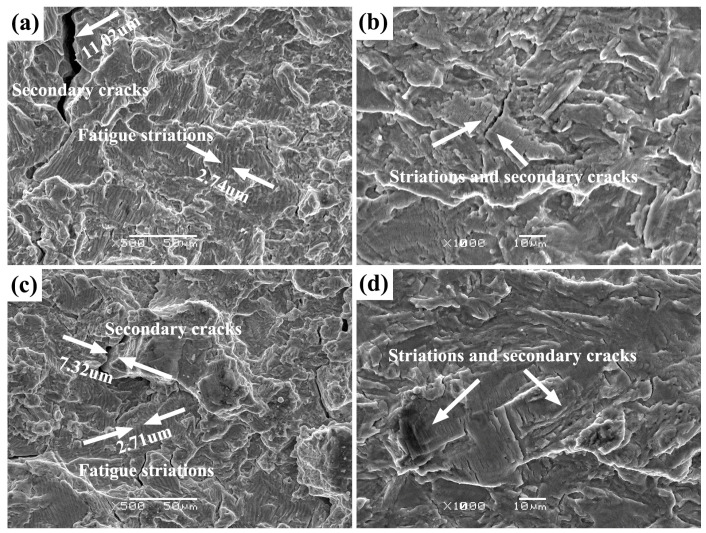
Fractographies of Zone II: (**a**,**b**) TC4-F alloy; (**c**,**d**) TC4 ELI alloy.

As Δ*K* increases, the crack propagation rate also increases. The plastic zone at the crack tip will pass through multiple grains, and the crack begins to propagate simultaneously or alternately along multiple slip systems. Therefore, as shown in Figure 7, the fracture morphologies of TC4-F and TC4 ELI alloys are consistent, and mainly consist of secondary cracks and fatigue striations. The figure shows a mixed mode of transgranular and intergranular fracture. Moreover, the secondary crack and fatigue striation are perpendicular to the propagation direction of the main crack. For TC4-F alloy, the fatigue striation spacing is approximately 2.74 μm, and the maximum secondary crack size is 11.02 μm (Figure 7a), which are larger than that of TC4 ELI alloy (fatigue striation spacing approximately 2.71 μm, secondary crack size about 7.32 μm, Figure 7c). There are numerous explanations and studies on fatigue striations. Currently, the model proposed by Larid [28], which described the plastic blunting of the crack tip, is widely accepted. It means that plastic blunting of the crack tip could cause the fatigue crack to move forward a certain distance in each cycle.

In this work, the used stress ratio R of 0.1 leads to absence of reverse compressive stress, which decreases the closing effect of fatigue cracks, and so the blunting of the crack tip caused by plastic deformation cannot be fully achieved. But Elber [29] thought that even tensile loads could also cause fatigue cracks to close, and proposed a mechanism for crack closure induced by plasticity. When the crack propagates forward through the plastic zone near the tip, only elastic recovery occurs near the new fracture surface. The residual plastic deformation cannot be restored, and can only be left behind. Under the cyclic loading, the stress intensity factor and the size of the plastic zone at the crack tip will gradually increase, which leads to the formation of a “plastic envelope zone” behind the plastic tip of the material that has already undergone permanent deformation. As a result, the opening displacement of the crack is effectively shortened, and the crack surface can also close.

It can be seen from Figure 7b,d that fatigue striations are mostly concentrated in the smaller grains, which indicates that the deformation of the duplex microstructure samples under cyclic loading mainly occurs in α_p_ [30]. Some cross cracks can also be observed on the figure, which may be caused by the initiation of dislocations in the β transformation structure. In addition, it can be observed that the orientation of fatigue striations in different regions is different on the fracture morphology. The microstructure characteristics in the process of crack propagation may cause the directional change of local fatigue striations. The fatigue striations formed during crack opening and closing should theoretically correspond to the number of cycles. Although crack propagation may not always form fatigue striations, the crack propagation rate can also be compared and analyzed by the spacing of fatigue striations. Therefore, the fatigue striation spacing can represent the crack propagation rate [18,31]. The fatigue striation-spacing difference between TC4-F alloy and TC4 ELI alloy is very small (Figure 7a,c), indicating that the FCP rates of the two alloys are almost coincident in the steady propagation stage, which is consistent with the results obtained in Figure 3.

(3) Zone III

**Figure 8:**
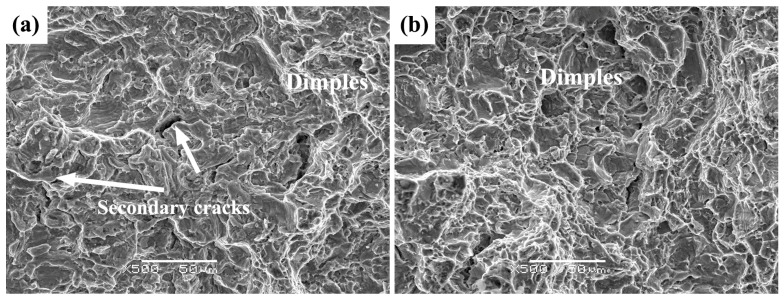
Fractography of Zone III: (**a**) TC4-F alloy; (**b**) TC4 ELI alloy.

During this stage, under the condition of very high Δ*K*, the fatigue crack begins to propagate rapidly, until the sample fails and breaks. Figure 8 shows the morphology of zone III of the TC4-F and TC4 ELI alloys. The fractographies of the two alloys are almost identical, mainly composed of dimples and secondary cracks, showing a microporous aggregation fracture mechanism.

### 3.4. FCP Path of TC4-F Alloy

**Figure 9:**
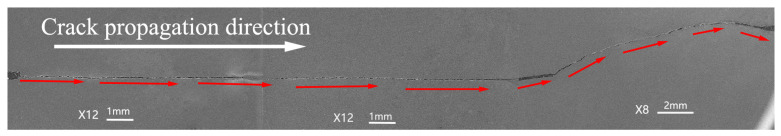
Partial crack-growth path of TC4-F alloy.

Shown in Figure 9 is the crack propagation path of the TC4-F alloy sample after failure in the FCP test. From the figure, it can be observed that from the initiation of the crack to the rapid propagation stage of the crack, the propagation path remains straight, without an obvious phenomenon of crack branching during this process. The main crack is almost moving along the direction perpendicular to the stress loading.

**Figure 10:**
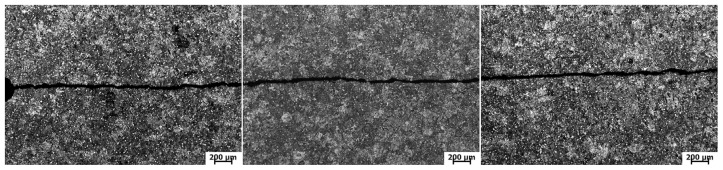
Crack propagation-path profiles of early crack-propagation region for TC4-F alloy.

Figure 10 illustrates the profile of the early crack propagation path of the TC4-F alloy sample near the pre-crack region. At the initial stage of cyclic loading, a sharp stress concentration at the notch root leads to the preferential nucleation of cracks. There is a certain angle between the propagation direction of the main crack and the loading stress. It is mainly because the crack is most likely to initiate and propagate on the slip surface with the maximum shear stress.

**Figure 11:**
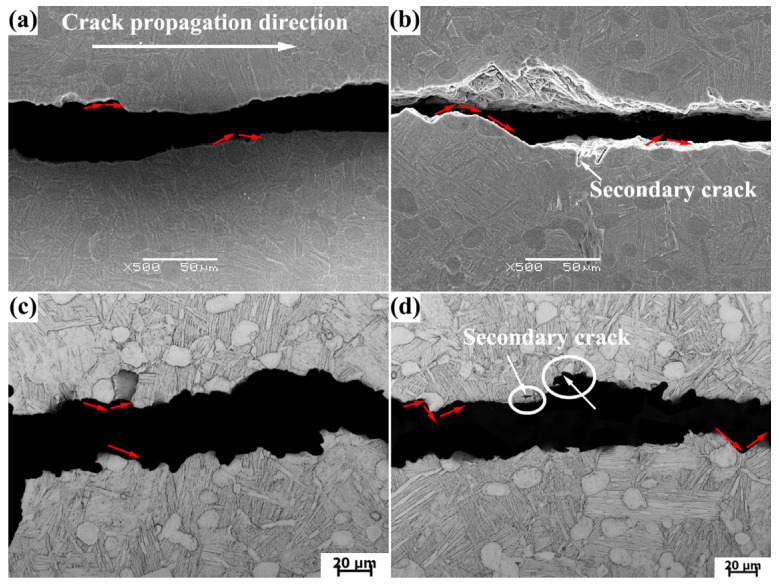
Crack early propagation of TC4-F alloy: (**a**,**b**) SEM; (**c**,**d**) optical microscope.

Based on Figure 10, the microscopic morphological characteristics of the crack initiation and the early crack-propagation zone as shown in Figure 11 were captured. It can be found that the cracks mainly initiate at the interface between the α_p_ phase and the β phase. Under the applied stress, the cracks gradually become larger, and propagate by “bypassing” and “cutting” the α_p_ phase (as indicated by the red arrows). The latter has little effect on the propagation rate of the main crack. However, when cracks propagate in the way of bypassing the α_p_ phase, the propagation path is prolonged and part of the energy is consumed, which can effectively reduce the propagation rate of the main crack. Small secondary cracks were also observed in the vicinity of the main crack-propagation path (Figure 11b,d), which is caused by the deformation incompatibility caused by the plastic inhomogeneity between α/β lamellae. As shown in Figure 11d, the phenomenon of crack deflection along the α_p_ phase boundary can be also observed. The small micro-cracks that do not propagate and crack deflection in the β-transformed microstructure can consume a certain amount of energy and reduce the local driving force at the crack tip [32], which can effectively slow down the crack growth rate.

## 4. Discussion

To sum up, the differences in crack propagation behavior between TC4-F and TC4 ELI alloys only lie in the early crack-propagation rate and morphology (∆*K* < 12.75 MPa·m^1/2^). Obviously, the crack propagation rate and morphology are determined by the type of alloys, which dramatically controls the microstructure and depends on the chemical composition. In this work, the Fe element mainly contributes to the differences in chemical composition between TC4-F and TC4 ELI alloys. In addition, during crack propagation, plastic deformation occurring at the crack tip could lead to the formation of a butterfly-shaped plastic deformation zone [33]. It has been reported that the FCP rate is mainly determined by the size of the plastic deformation zone [34].

The calculation formula for the plastic deformation zone in the plane stress state can be given as follows [9,31]:

(4) $$r_{p}=\frac{1}{\pi }{\left(\frac{∆K}{\sigma_{y}^{\prime }}\right)}^{2}$$

where $\sigma_{y}^{\prime }$ is the cyclic yield strength, which can be replaced by monotonic yield strength (*σ*_y_) [35]. Research has suggested that both larger plastic zones at crack tips and rough fatigue crack sections can enhance the degree of crack closure, reducing the crack propagation rate [36,37]. According to the point A in Figure 3, when ∆*K* = 12.75 MPa·m^1/2^, the plastic zone size of TC4-F and TC4 ELI alloys can be calculated to be 5.52 μm and 6.06 μm, respectively. By contrast, the difference in plastic zone size between them is only 0.54 μm. This means that when ∆*K* < 12.75 MPa·m^1/2^, the plastic zone size difference between the TC4-F and TC4 ELI alloys turns out to be even smaller (<0.54 μm), which may have little influence on the FCP rate of the two alloys.

The ∆*K* at the stage of crack initiation and early propagation is very small, and the crack propagation occurs between several grains. Due to the different orientations between adjacent grains, the crack needs to break the grain boundary to propagate [38]. The existence of grain boundaries increases the resistance to crack propagation and even restricts the cracks to the grain boundaries. Crack propagation within grains mainly occurs at the slip plane (a closely packed plane), where resistance is least. Therefore, grain boundary obstruction is the key to crack propagation. If the crack is suppressed on the grain boundary before entering the adjacent grains, the stress will accumulate on the grain boundary. When the accumulated stress can induce the activation of different slip systems in the grains, this process will change the crack propagation path. When the accumulated stress is large enough to activate multiple slip systems, the crack will no longer propagate along the plane, thus increasing the roughness of the fracture surface [39]. This increases the resistance for cracks to propagate from the grain boundaries, and requires more cycles for propagation.

**Figure 12:**
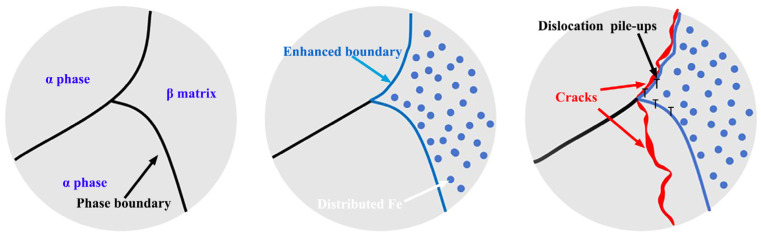
The schematic diagram of the effect of adding Fe.

Moreover, working as a β-stable element, the trace Fe added in TC4-F alloy and homogeneously distributed in the β matrix along the α_p_/β or α_s_/β boundary could enhance the α/β boundary resistance. Under cyclic loading, the activated dislocations in the α phase during deformation move to the α/β boundary. Due to the crystallographic misorientation and lattice incompatibility, the continued motion of these dislocations could be impeded by the boundaries and pile up at the boundary front, creating a localized stress concentration. For titanium alloy with duplex microstructure, microcracks preferentially nucleate at stress concentrations along the boundaries, and subsequently coalesce to form long cracks propagating along the phase boundaries or through the α_p_ phase under cyclic loading [16]. But the increased boundary resistance makes it more difficult for activated dislocations to escape, increasing the accommodation for the accumulation of dislocation. Meanwhile, the pinning effect of Fe atoms restricts the dislocation movement. Figure 12 gives the schematic diagram of the effect of adding Fe. Theses mechanisms above effectively mitigate stress concentration, thereby delaying the initiation and growth of the microcrack. It means that, compared with the initiation of cracks of TC4 ELI alloy, more energy consumption (higher stress) is needed for TC4-F alloy to overcome the boundary resistance. And the behavior of long cracks propagating along the phase boundaries or through the α_p_ phase also needs extra stress. Therefore, the crack could be inhibited on the phase boundary in the early propagation (Figure 3), with the accumulation of stress and the non-planar propagation of the crack occurring, increasing the roughness of the section and resulting in a lower crack-propagation rate of TC4-F alloy. But, with the continuous loading, the effect of Fe described above turns out to be negligible, once the cracks start to propagate rapidly. Moreover, the way of propagating by bypassing the α_p_ phase increases the crack propagation path (Figure 11), which is also conducive to reducing the rate of crack propagation for TC4-F alloy.

## 5. Conclusions

In this work, based on the fatigue crack propagation-rate curves, the corresponding fracture morphologies of different crack propagation stages, and the effect of Fe on crack propagation for TC4-F alloy with duplex microstructure, were investigated in detail, and compared with the TC4 ELI alloy. Conclusions can be drawn, as follows:

(1) When ∆*K* < 12.75 MPa⋅m^1/2^, the d*a*/d*N* curves show that TC4-F owns lower fatigue crack-propagation rate. But when ∆*K* > 12.75 MPa⋅m^1/2^, the d*a*/d*N* curves of the two alloys are almost overlapping.
(2) The fracture could be divided into three parts, reflecting the different stages of crack propagation. And the main differences in fracture characteristics between TC4-F and TC4 ELI alloys mainly focus on Zone I, showing a rough section.
(3) The addition of trace Fe plays an important role in α/β boundary resistance, reducing the crack growth rate during the stage of crack initiation and early propagation. Also, crack propagating by bypassing the α_p_ phase also helps to reduce the propagation rate.

## Figures and Tables

**Table 1:** Chemical composition of the investigated alloys (in wt. %).

	Al	V	Fe	C	N	O	H	Ti
TC4-F	6.20	4.14	0.537	0.020	0.020	0.13	0.001	Bal.
TCE ELI	6.20	4.28	0.076	0.010	0.017	0.09	0.002	Bal.

## Data Availability

The original contributions presented in this study are included in the article. Further inquiries can be directed to the corresponding author.
